# Does the frequency and intensity of physical activity in adolescence have an impact on bone? The Tromsø Study, *Fit Futures*

**DOI:** 10.1186/s13102-015-0020-y

**Published:** 2015-11-10

**Authors:** Tore Christoffersen, Anne Winther, Ole Andreas Nilsen, Luai Awad Ahmed, Anne-Sofie Furberg, Guri Grimnes, Elaine Dennison, Nina Emaus

**Affiliations:** 1 Department of Health and Care Sciences, UIT The Arctic University of Norway, Forskningsparken, Sykehusveien 21, NO-9037 Tromsø, Norway; 2 Finnmark Hospital Trust, Alta, Norway; 3 Division of Neurosciences, Orthopedics and Rehabilitation Services, University Hospital of North Norway, Tromsø, Norway; 4 Institute of Public Health, College of Medicine and Health Sciences, United Arab Emirates University, Al Ain, UAE; 5 Department of Community Medicine, UIT the Arctic University of Norway, Tromsø, Norway; 6 Division of Internal Medicine, University Hospital of North Norway, Tromsø, Norway; 7 Tromsø Endocrine Research Group, Department of Clinical Medicine, UiT The Arctic University of Norway, Tromsø, Norway; 8 MRC Lifecourse Epidemiology Unit, Southampton, UK; 9 Victoria University, Wellington, New Zealand

**Keywords:** Population-based study, Physical activity, Adolescents, Bone mineral density, DXA

## Abstract

**Background:**

Optimization of the genetic potential for bone accrual in early life may prevent future fractures. Possible modification factors include lifestyle factors such as nutrition and physical activity. Measured levels of bone mineral density (BMD) and bone mass content (BMC) are indicators of bone strength, and are correlated with fracture risk. This study explored the impact of self-reported physical activity frequencies and intensity on BMD and BMC in Norwegian adolescents.

**Methods:**

In 2010–2011 school students in two North-Norwegian municipalities were invited to a health survey, the Fit Future study. 508 girls and 530 boys aged 15–18 years attended. BMD and BMC were measured by dual X-ray absorptiometry. Physical activity and other lifestyle-factors were reported by questionnaires and clinical interviews. Statistical analyses were performed sex stratified, using ANOVA for comparison of means and linear regression models adjusting for factors known to affect bone.

**Results:**

Approximately 2/3 of girls and boys reported themselves as physically active outside school hours. Active participants had a significantly higher BMD and BMC at all sites (*p* < 0.001), except for BMC total body in girls, compared to inactive participants. In multiple linear regression analyses, increased physical activity measured as days a week, categorized into seldom, moderate and highly, was positively associated with BMD (g/cm^2^) at all sites in girls. Girls reporting themselves as highly active had BMD levels 0.093 g/cm^2^, 0.090 g/cm^2^ and 0.046 g/cm^2^ higher (*p* < 0.001) than their more seldom active peers at femoral neck, total hip and total body respectively. Corresponding values for boys were 0.125 g/cm^2^, 0.133 g/cm^2^ and 0.66 g/cm^2^. BMC measures showed similar trends at femoral neck and total hip.

**Conclusions:**

Increased level of physical activity is associated with higher BMD and BMC levels in adolescents. For both sexes high activity frequency seems to be essential, whilst boys reporting quite hard intensity has an additional impact. The differential effects of physical activity on bone strength in adolescence have clinical implications, especially in preventive strategies.

## Background

Increased bone fragility leads to higher fracture risk and osteoporotic fractures are a major health issue worldwide [1–3]. Amongst the Scandinavian countries, Norway has the highest incidences of forearm and hip fractures ever reported [4]. While traditional preventive strategies has focused on age-related bone loss and frequencies of fracture among the elderly, attention has recently focused towards the contribution of peak bone mass (PBM) on bone strength [5]. Evidence indicates that optimization of the genetic potential for bone accrual in the first few decades of life can reduce later risk of osteoporosis [6]. This optimization includes modification of lifestyle factors such as nutrition and physical activity levels.

Physical activity is an important and relevant preventive factor with its potential to improve strength, flexibility, coordination, balance, reaction time and endurance, as well as for its ability to modify bone loss through changes in bone structure and geometry by reaction to mechanical stress on bone [7]. The mechanostat theory suggests that dynamic loads are essential for bone adaptation [8]. Muscular work during physical activity provides load on the skeleton, and the evidence of physical activity’s influence on bone structure is strong, both during childhood, adolescent and adulthood [7]. The establishment of habitual physical activity during childhood and adolescent is especially important, as the mechanical loading benefits seem particularly strong during growth and established habits may last during later life [6, 9]. In contrast, immobilization studies show that removal of dynamic loading results in loss of bone mass followed by changes in structural parameters [10, 11]. Establishing physical activity levels during growth and maintaining these levels through life are therefore crucial to bone strength. However, the levels of frequencies and intensity that are required for optimal stimulation of bone are still a matter of discussion.

Several studies on effect of physical activity on bone have investigated bone mass in athletic individuals preforming specific sports with repetitive impact [12–17]. In a normal population, where the same individual may practice a variety of activities at more or less structured programs, and maybe at random periods in time, comparatively less is known of the impact of activity on bone. Even though recent cohort studies have focused on measuring physical activity levels at different sexes and ages [18–21], clinical applicable guidelines based on different levels of physical activity, concerning different anatomical sites, is still in need of evidence. In particular during the potent years of adolescent where habits and lifestyle are exposed to changes in autonomy.

The main objective of this study was to explore the relationship between self-reported physical activity and bone mineral density (BMD) and bone mineral content (BMC) levels. Furthermore, we wanted to identify how different quantities of activity influence BMD and BMC levels in this convenience sample of Norwegian adolescents.

## Methods

### Study population and design: Fit Futures

We have previously described in details the Fit Futures participants and the recruitment procedures [22]. In brief, the Fit Futures study is an expansion of the Tromsø study which invited all first-year upper-secondary school students in the urban municipality Tromsø and the more rural neighbor municipality Balsfjord to take part in a population based cross-sectional study. Information about the study was given in classrooms and through the schools web sites. In total, 1117 participants were invited, and 1,038 (508 girls and 530 boys) attended the survey giving an attendance rate of 92.9 %. Dedicated research technicians in a well-established research unit at the University Hospital of North Norway (UNN) ran all examinations during the school day. All participants gave written informed consent. Participants younger than 16 years of age signed with written permission from guardians and individuals aged 16 and above signed at the study site. The study was approved by The Norwegian Data Protection Authority (reference number 2009/1282) and by The Regional Committee of Medical and Health Research Ethics (reference number 2011/1702/REKnord).

### Measurements

Height and weight were measured in all participants after standardized procedures including light clothing and no shoes on an automatic electronic scale, Jenix DS 102 stadiometer (Dong Sahn Jenix, Seoul, Korea). BMI was calculated as weight in kilograms divided by the squared height in meters. In all participants, bone parameters were measured at the total hip, the femoral neck and total body by dual X-ray absorptiometry (DXA; GE Lunar prodigy, Lunar Corporation, Madison, WI, USA) using the enCORE pediatric software [23]. The same devise was used throughout the entire study. Ten scans were excluded after quality control. The densitometer coefficient of variation has been estimated to 1.17 % for total hip and 1.72 % for femoral neck [24], whereas CV for total body has not been calculated.

### Questionnaires

All information regarding lifestyle factors was collected using self-reporting electronic questionnaires. Past medical history and alternatively use of medication, including contraceptives, were recorded through a clinical interview. The DXA lab technicians registered ethnicity and excluded participants with a possible pregnancy.

The explorations of physical activity levels were categorized throughout several validated questions [25]. First the participants were asked the question “Are you actively doing sports or physical activity outside school hours?” dividing them in groups of active (“yes”) or inactive (“no”). Physical activity frequencies were determined by “If you are actively doing sports or physical activity outside school, how many days a week are you active?” and categorized into “never”[1], “less than once a week”[2], “1 day a week”[3],”2 to 3 days a week”[4], “4 to 6 days a week”[5] and “almost every day”[6]. Perceived intensity of physical activity was categorized in 5 groups, namely: not hard at all [1], a bit hard [2], quite hard [3], very hard [4] and extremely hard [5]. The answers on physical activity frequencies were recoded into three possible groups. For this questions the answers [1] and [2] were called seldom, [3] and [4] were called moderate, and [5] and [6] highly. The answers on perceived intensity were divided into not hard [1–2], quite hard [3] and hard [4–5]. Additional measurements for fitness was not available.

### Statistical analyses

Statistical analyses were performed sex stratified. Continuous variables were described as mean and standard deviation, while categorical variables were described by numbers and percentages. Differences in BMD and BMC levels between the inactive and the active groups were tested using student *t*-test. To study any correlation between ordinal categorical groups, Spearman`s correlation coefficient were used.

ANOVA was used to assess the differences in BMD and BMC according to the different levels of physical activity and intensity. Levene`s test were used to control for homogeneity of variance between groups, followed by Bonferroni post hoc test for multiple comparisons within the groups. If there were doubt about homogeneity of variance, Games-Howell procedure was used.

We further performed simple linear regression for BMD and BMC levels using femoral neck, total hip and total body as anatomical sites. Variables contributing at 10 % significance level such as age, height, weight, sexual maturation, smoking, alcohol consumption (in boys), hormonal contraceptives (in girls), diseases and medication known to affect bone together with different physical activity groups were used for multiple regression analyses. Two models were used in multiple regression analyses. The first including anthropometric variables such as age, height, weight. Further model 2 included lifestyle variables such as sexual maturation, smoking and alcohol intake. We used residual analyses to check the normal distribution, linearity, homogeneity of variance and outliers. Excluding participants older than 18 years of age gave no changes to the findings. No assumptions were considered violated. Logistic regression was used to calculate odd ratios when relevant. The analyses were performed using the Statistical Package of Social Science (SPSS v. 22) and all values of *p* < 0.05 were considered statistical significant.

## Results

The main characteristics of the participants are shown in Table 1. Mean age (SD) for menarche was 12.95 years (1.19). For most of the boys, sexual maturation was categorized as “underway”. In total, 98 % answered the questions on smoking and snuffing with 23 % reported smoking sometimes or daily whilst 37 % reported snuffing. Lifestyle variables, except smoking, were significantly differently distributed between the sexes (*p* < 0.005). Among the girls 66 % reported themselves as physical active outside school hours, whilst 65 % of the boys reported the same.

**Table 1:** Baseline characteristics and bone mineral density (BMD) and contents (BMC) levels at different sites by status of physical activity outside school hours. The Tromsø Study, *Fit Futures*

	Inactive		Active		Mean Δ (Choen`s d)
	N	Mean (SD)	N	Mean (SD)	
Girls					
Age (years)	166	16.6 (1.5)	333	16.3 (1.2)	
Height (cm)	164	163.7 (7.00)	333	165.2 (6.36)	
Weight (kg)	164	61.4 (13.4)	333	61.3 (11.6)	
BMI (kg/m^2^)	164	22.9 (4.5)	333	22.5 (4.1)	
Smoking	166		332		
*Daily*		*12.7 %*		*2.1 %*	
*Sometimes*		*20.6 %*		*13.9 %*	
*No, never*		*66.7 %*		*84.0 %*	
Alcohol	166		333		
*Never*		*23.5 %*		*24.0 %*	
*≤1/month*		*38.6 %*		*48.9 %*	
*≥2/month*		*37.9 %*		*27.1 %*	
FN-BMD (g/cm^2^)	156	1.030 (0.113)	331	1.084 (0.124)	***−0.054*** (0.53)
TH-BMD (g/cm^2^)	151	1.025 (0.112)	329	1.077 (0.127)	***−0.053*** (0.51)
TB-BMD (g/cm^2^)	163	1.125 (0.076)	332	1.151 (0.076)	***−0.026*** (0.40)
FN-BMC (g)	156	4.727 (0.662)	331	5.008 (0.714)	***−0.282*** (0.47)
TH-BMC (g)	151	30.79 (4.63)	329	32.61 (4.89)	***−1.820*** (0.45)
TB-BMC (g)	163	2493.0 (439.6)	332	2555.4 (381.3)	−62.40 (0.18)
Boys					
Age (years)	177	16.2 (0.8)	343	16.3 (1.2)	
Height (cm)	177	176.3 (6.8)	343	177.3 (6.5)	
Weight (kg)	177	71.4 (17.3)	343	70.2 (13.0)	
BMI (kg/m^2^)	177	22.9 (5.1)	343	22.3 (3.7)	
Smoking	177		343		
*Daily*		*6.2 %*		*2.6 %*	
*Sometimes*		*27.1 %*		*17.2 %*	
*No, never*		*66.7 %*		*80.2 %*	
Alcohol	175		343		
*Never*		*31.4 %*		*30.9 %*	
*≤1/month*		*33.1 %*		*39.4 %*	
*≥2/month*		*35.4 %*		*29.8 %*	
FN-BMD (g/cm^2^)	173	1.057 (0.140)	339	1.129 (0.150)	***−0.072*** (0.56)
TH-BMD (g/cm^2^)	170	1.064 (0.140)	333	1.142 (0.147)	***−0.078*** (0.62)
TB-BMD (g/cm^2^)	175	1.157 (0.097)	342	1.195 (0.094)	***−0.037*** (0.45)
FN-BMC (g)	173	5.639 (0.871)	339	6.147 (1.019)	***−0.508*** (0.59)
TH-BMC (g)	170	37.83 (6.13)	333	41.06 (6.60)	***−3.239*** (0.57)
TB-BMC (g)	175	2862.8 (461.8)	342	3020.7 (479.3)	***−157.9*** (0.38)

Values significant at *p* < 0.001 in Italic Bold

The mean BMD in girls reporting themselves to be active outside school hours was significantly higher at all sites compared to their inactive counterparts (*p* < 0.001) (Table 1). The active girls also had a higher BMC (SD) at femoral neck and total hip (*p* < 0.001), whilst the mean difference at total body was not significantly different (*p* = 0.105). In boys, participants reported to be active outside school hours had higher BMD and BMC levels (*p* < 0.001) at all anatomical sites (Table 1).

**Fig. 1:**
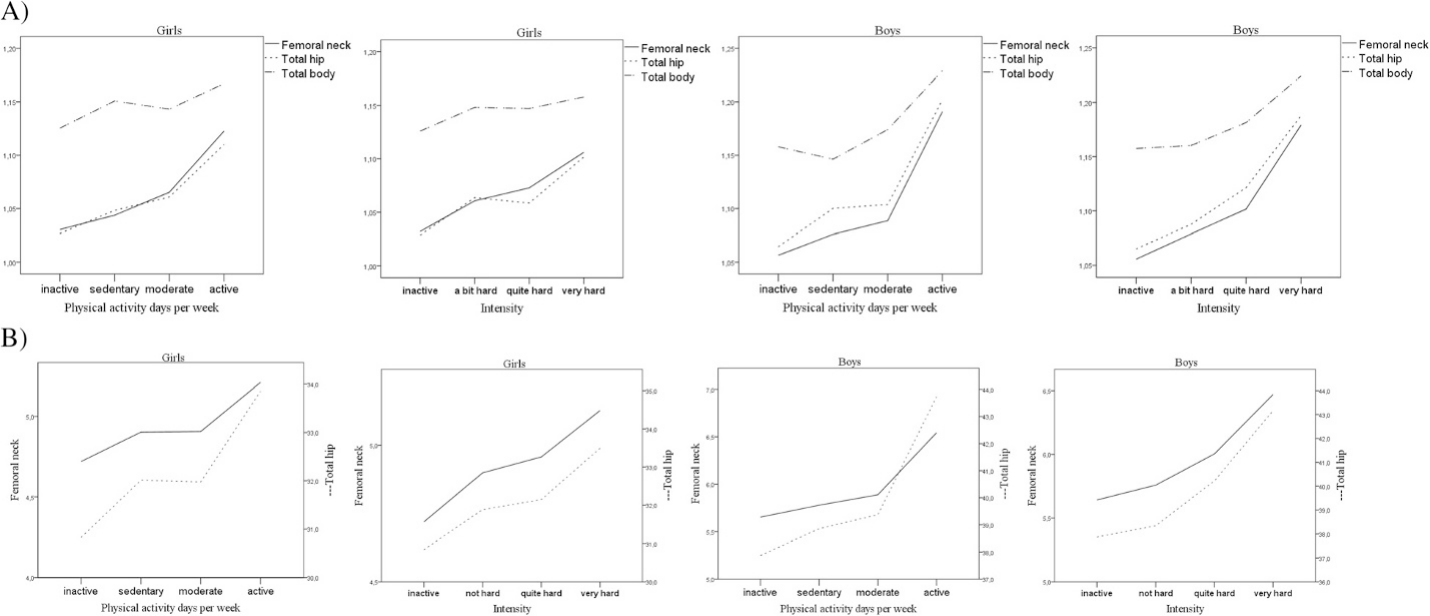
Bone mineral density in g/cm^2^ (**a**) and bone mineral content in g (**b**) at different anatomical sites by reported physical activity frequency and intensity outside school hours in Norwegian girls and boys

In Fig. 1a) the two left panels illustrates the trends of increasing BMD levels at higher self- reported physical activity level as days per week (*p* ≤ 0.001) and intensity (*p* ≤ 0.001) in girls. The two right panels show the same statistical significant linear trends for boys in both frequencies (*p* < 0.001) and intensity (*p* < 0.001). Correspondingly, Fig. 1 B) illustrates increasing BMC levels in femoral neck and total hip at higher physical activity frequencies (*p* < 0.001) and intensities (*p* < 0.001) for both sexes. BMC in total body showed statistical significant linear trends both for frequencies (*p* < 0.001) and intensity (*p* < 0.001) only in boys (data not shown).

In multivariate analyses, increasing physical activity levels reported as days a week were positively associated with BMD and BMC in both sexes, in models including age, height and weight. Girls that reported being moderately active had higher BMD and BMC levels at femoral neck compared to girls who reported themselves as being seldom active (*p* < 0.05), whilst girls reporting high frequencies of training increased BMD and BMC at femoral neck even further (Table 2). BMC levels at total hip and total body were not significantly increased in girls reported moderate activity compared to those reported seldom activity, but the highly active group had a 2.879 g (*p* < 0.001) and 98.972 g (*p* < 0.05) increase at these sites respectively. Boys who reported themselves highly active had BMD levels 0.125 g/cm^2^, 0.133 g/cm^2^ and 0.066 g/cm^2^ higher (*p* < 0.001) than their more seldom active peers at femoral neck, total hip and total body respectively. BMC levels increased comparing seldom to moderate (*p* < 0.05) and seldom to highly active boys (*p* < 0.001) at all sites (Table 3).

**Table 2:** Associations between levels of physical activity outside school hours and bone mineral density and content in Norwegian girls 15–18 years. The Tromsø Study, *Fit Futures*

	BMD (β coefficient [95 % CI])			BMC (β coefficient [95 % CI])		
	Femoral neck	Total hip	Total body	Femoral neck	Total hip	Total body
Physical Activity Frequency (Days a week)						
*Model 1*						
Seldom	Reference					
Moderate	*0.031* [0.008, 0.055]	*0.033* [0.009, 0.057]	*0.016* [0.003, 0.029]	*0.138* [0.020, 0.255]	0.789 [−0.011, 1.589]	18.901 [−30.260, 68.062]
High	***0.093*** [0.066, 0.121]	***0.090*** [0.062, 0.117]	***0.046*** [0.031, 0.062]	***0.477*** [0.339, 0.615]	***2.879*** [1.942, 3.816]	*98.972* [41.055, 156.889]
*Model 2*						
Seldom	Reference					
Moderate	*0.026* [0.002, 0.050]	*0.028* [0.004, 0.052]	0.012 [−0.001, 0.026]	0.094 [−0.025, 0.213]	0.671 [−0.154, 1.495]	7.138 [−43.040, 57.316]
High	***0.094*** [0.066, 0.122]	***0.092*** [0.063, 0.121]	***0.048*** [0.033, 0.064]	***0.464*** [0.322, 0.606]	***2.961*** [1.980, 3.942]	***100.618*** [40.517,160.718]
Physical Activity Intensity						
*Model 1*						
Not hard	Reference					
Quite hard	*0.036* [0.011, 0.061]	*0.029* [0.004, 0.054]	*0.022* [0.008, 0.036]	*0.190* [0.064, 0.316]	*1.012* [0.161, 1.862]	39.536 [−12.725, 91.798]
Hard	***0.066*** [0.041, 0.091]	***0.069*** [0.043, 0.094]	***0.031*** [0.017, 0.046]	***0.339*** [0.213, 0.466]	***2.170*** [1.314, 3.027]	51.061 [−1.817, 103.940]
*Model 2*						
Not hard	Reference					
Quite hard	*0.031* [0.006, 0.057]	0.026 [0.000, 0.52]	*0.018* [0.004, 0.032]	*0.149* [0.021, 0.277]	*0.966* [0.085, 1.846]	29.257 [−24.215, 82.729]
Hard	***0.058*** [0.032, 0.084]	***0.062*** [0.035, 0.088]	***0.026*** [0.011, 0.040]	***0.285*** [0.155, 0.416]	***2.029*** [1.135, 2.922]	34.902 [−19.689, 89.494]

Model 1: adjusted for age, weight, height

Model 2: Model 1 + sexual maturation, smoking status, alcohol intake

Values significant at *p* < 0.05 in Italic, values significant at *p* < 0.001 in Italic Bold

**Table 3:** Associations between levels of physical activity outside school hours and bone mineral density and content in Norwegian boys 15–18 years. The Tromsø Study, *Fit Futures*

	BMD (β coefficient [95 % CI])			BMC (β coefficient [95 % CI])		
	Femoral neck	Total hip	Total body	Femoral neck	Total hip	Total body
Physical Activity Frequency (Days a week)						
*Model 1*						
Seldom	Reference					
Moderate	*0.034* [0.008, 0.060]	*0.041* [0.015, 0.068]	*0.019* [0.003, 0.034]	*0.241* [0.077, 0.404]	*1.652* [0.588, 2.716]	*65.921* [7.096, 124.747]
High	***0.125*** [0.096, 0.154]	***0.133*** [0.103, 0.162]	***0.066*** [0.049, 0.083]	***0.779*** [0.599, 0.959]	***5.134*** [3.962, 6.306]	***234.404*** [169.38, 299.425]
*Model 2*						
Seldom	Reference					
Moderate	*0.029* [0.002, 0.056]	*0.037* [0.010, 0.064]	*0.017* [0.001, 0.033]	*0.220* [0.054, 0.386]	*1.544* [0.463, 2.626]	*59.838* [−0.095, 119.771]
High	***0.125*** [0.095, 0.155]	***0.132*** [0.102, 0.161]	***0.067*** [0.049, 0.084]	***0.797*** [0.613, 0.980]	***5.172*** [3.976, 6.368]	***241.298*** [174.753, 307.843]
Physical Activity Intensity						
*Model 1*						
Not hard	Reference					
Quite hard	*0.042* [0.014, 0.070]	***0.052*** [0.024, 0.080]	*0.023* [0.007, 0.040]	*0.311* [0.136, 0.486]	*2.068* [0.925, 3.212]	*85.706* [23.172, 148.241]
Hard	***0.109*** [0.081, 0.137]	***0.113*** [0.085, 0.141]	***0.058*** [0.042, 0.075]	***0.671*** [0.497, 0.845]	***4.302*** [3.166, 5.438]	***209.455*** [146.958, 271.952]
*Model 2*						
Not hard	Reference					
Quite hard	*0.033* [0.005, 0.061]	*0.045* [0.017, 0.074]	*0.020* [0.003, 0.036]	*0.266* [0.091, 0.442]	*1.817* [0.674, 2.959]	*73.031* [10.216, 135.846]
Hard	***0.105*** [0.076, 0.133]	***0.109*** [0.080, 0.137]	***0.055*** [0.038, 0.072]	***0.658*** [0.481, 0.835]	***4.191*** [3.043, 5.340]	***199.326*** [136.122, 262.529]

Model 1: adjusted for age, weight, height

Model 2: Model 1 + sexual maturation, smoking status, alcohol intake

Values significant at *p* < 0.05 in Italic, values significant at *p* < 0.001 in Italic Bold

Comparing low intensity training to medium and hard training gave positive associations at all sites in girls, except at total body BMC (Table 2). Also the boys had positive regression coefficients in comparison of low intensity training boys with those training quite to extremely hard (Table 3). Including lifestyle factors (sexual maturation, smoking and use of alcohol) in a second model only made minor changes in both sexes on the association between physical activities levels and bone measures.

## Discussion

Physical activity is established as an important contributor for bone mass accrual [7], and this study supports the suggestion that there is a dose response relationship between activity and bone mass. In Norwegian adolescents, 2/3 reported themselves to be active outside school and already had a possible head start in reaching an optimal peak bone mass compared to participants living a more sedentary life. Furthermore, among the active youths, high levels of activity frequencies and intensity appeared to enlarge the effect on BMD as well as BMC levels in both sexes. Even when adjusting for possible confounding factors, both anthropometric and lifestyle, known to be significantly associated with bone mass, these effects were preserved. Girls being highly active several days a week had the highest bone mass levels, however, also moderate activity seemed beneficial. The boys had an additional effect of training quite and extremely hard. There was a significant linear trend at all sites. With BMD and BMC levels at femoral neck and total hip 10-14 % above seldom active adolescents, and even higher above the inactive participants, the highly active youths have gained close to 1 SD higher level of bone mass in almost every measured site. Although debated, Rizzoli et al. have calculated that a 10 % increase in peak bone mass corresponds to a 1 SD BMD gain in adulthood, or, a fracture risk reduction of nearly 50 % [5].

The mechanostat theory suggest how bones adapt their strength to the mechanical loads exerted on them [8]. Physical activity produces dynamic loads that can stimulate bone mass, geometry and architecture. The findings in the presents study among adolescents correspond to previously reported dose–response results in both the same [22] and other cohorts [26–28]. From the ALSPAC cohort, Deere et al. reported positive associations between femoral neck BMD and high impact activity [26] in both sexes. With a slightly older cohort, with otherwise comparable characteristics, their results with beta values of 0.096 for femoral neck BMD at impacts above 4.2 gravitational forces, measured with accelerometer, corresponds to our beta value of 0.105 in boys reporting very hard intensities during physical activity. As Deere et al. used an accelerometer to define impact; the comparability strengthens the suggestion of a strong positive association between vigorous activity and bone mineral measurements in our findings. Furthermore, reports from the PBMAS cohort categorizing adolescents as inactive, average and active, show greater adjusted bone geometry measures at the proximal femur [27]. The same cohort has previously been described through the same activity categorization with 8-11 % higher BMC in active participants at total body and total hip [29]. These differences are comparable to our 5-12 % increased BMC in active adolescents and reinforce the positive relation between adolescent bone strength and physical activity. Although bone properties measured with DXA is a common surrogate of bone strength, other methods of bone strength evaluation are possible to use. Three-dimensional imaging techniques open for quantification of bone geometry, architecture and volumetric density, and how these structural properties contribute to bone strength. In our study these methods were unfortunately not available. However findings from Daly and Bass [20] demonstrated that lifetime physical activity was associated with 6-15 % higher mid-femur total and cortical areas as measured by quantitative computed tomography (QCT), but no association between physical activity and areal BMD were found. Hence, with the two-dimensional aspect of DXA, the effect of physical activity loading on mechanical strength of bone may be underestimated in our study.

A recent study by Janz et al. [30], using accelerometers and a 12-year follow up, tested trajectories of physical activity for their effect on bone strength. Their results suggested that habitual high levels of physical activity not only at adolescence but also during childhood was associated with increased bone strength, including measurements of geometry at weight bearing sites. These indications of a sustained effect of early physical activity, as well as the role of physical activity throughout childhood and adolescence were not possible to explore in our study. However, several authors claim a tracking of physical activity as a behavioral process [31–33], which seems plausible in our cohort. Also the CHAMPS study –DK, although with a mean age of 11.5 years, reported a positive association between physical activity and bone traits [34]. BMC and BMD increased as the proportion of time spent in moderate to high level physical activity increased opposed to the inactive and lower level of activity. Our findings support this suggestion of a threshold level or dose–response effect, not only in intensity or impact, but valid for frequencies as well.

In a systematic review by Bielemann et al. the associations between physical activity during life and bone mineral content or density in young adults were evaluated [7]. They found a higher degree of positive association in males than in females, when physical activity measurements followed transition from adolescence to adult life. In our study there were strong positive associations in both sexes, although the boys seemed to have an additional effect of reporting vigorous activity. Hence, the indication from Bielemann et al. that weaker associations in females could be explained by lower participation in peak strain activities, may be compensated by sufficient activity regarding frequency.

The strengths of this study are the population-based design including high attendance in both sexes and representation of adolescents from both urban and rural regions. Furthermore the variety in activity reported through frequency and intensity and measurements of both BMC and BMD at a variety of anatomical sites strengthens the results. The main limitation is the cross-sectional design with its restricted causal inferences. Follow-up data of this cohort is not yet available. There are also limitations in the physical activity data, which may weaken the associations. These data are completely of self-reported nature, although with validated questionnaires, but it can be questioned how detailed the interpretation in terms of dosage and extent can be [35]. Furthermore, only BMC and BMD measurements were available in this study, and consequently no other measures of bone strength could be explored, neither could counts of bone formation markers.

To our knowledge, this study is the first to identify how different variations in physical activity influence bone mass accrual in Norwegian adolescents. Previously findings have indicated that current BMD levels in Norwegian youths are comparable with other European populations [22] although fracture risk in the Norwegian elderly are high. Physical activity differentiated to days a week and intensity are associated with higher BMD and BMC levels in both sexes. Furthermore, the findings from this study indicate the importance of being active close to every day for girls and in addition with high intensity for boys. This dose–response trend could be of relevance for recognizing youths in danger of not reaching their potential for peak bone mass and hence an increased risk of future fractures.

## Conclusions

In conclusion, approximately 2/3 of a Norwegian cohort reported themselves to be physically active outside school hours. Self-reported physical activity is positively associated with bone mass accrual and there seems to be a linear trend among activity categories. The everyday, vigorous active adolescents have a considerable head start in future fractures reduction compared to their more sedentary counterparts.
